# Increased CD45RA^+^FoxP3^low^ Regulatory T Cells with Impaired Suppressive Function in Patients with Systemic Lupus Erythematosus

**DOI:** 10.1371/journal.pone.0034662

**Published:** 2012-04-10

**Authors:** Xiujun Pan, Xiangliang Yuan, Yingxia Zheng, Weiwei Wang, Jianping Shan, Fujun Lin, Gengru Jiang, Yuan H. Yang, Die Wang, Dakang Xu, Lisong Shen

**Affiliations:** 1 Department of Clinical Laboratory, Xinhua Hospital, Shanghai Jiao Tong University School of Medicine, Shanghai, China; 2 Department of Nephrology, Xinhua Hospital, Shanghai Jiao Tong University School of Medicine, Shanghai, China; 3 Centre for Inflammatory Diseases, Department of Medicine, Monash University, Melbourne, Australia; 4 Monash Institute of Medical Research, Monash University, Melbourne, Australia; 5 College of Basic Medical Science, Harbin Medical University, China; 6 Institute of Ageing Research, Hangzhou Normal University School of Medicine, Hangzhou, China; Pavillon Kirmisson, France

## Abstract

**Background:**

The role of naturally occurring regulatory T cells (Treg) in the control of the development of systemic lupus erythematosus (SLE) has not been well defined. Therefore, we dissect the phenotypically heterogeneous CD4^+^FoxP3^+^ T cells into subpopulations during the dynamic SLE development.

**Methodlogy/Principal Findings:**

To evaluate the proliferative and suppressive capacities of different CD4^+^ T cell subgroups between active SLE patients and healthy donors, we employed CD45RA and CD25 as surface markers and carboxyfluorescein diacetatesuccinimidyl ester (CFSE) dilution assay. In addition, multiplex cytokines expression in active SLE patients was assessed using Luminex assay. Here, we showed a significant increase in the frequency of CD45RA^+^FoxP3^low^ naive Treg cells (nTreg cells) and CD45RA^−^FoxP3^low^ (non-Treg) cells in patients with active SLE. In active SLE patients, the increased proportions of CD45RA^+^FoxP3^low^ nTreg cells were positively correlated with the disease based on SLE disease activity index (SLEDAI) and the status of serum anti-dsDNA antibodies. We found that the surface marker combination of CD25^+^CD45RA^+^ can be used to defined CD45RA^+^FoxP3^low^ nTreg cells for functional assays, wherein nTreg cells from active SLE patients demonstrated defective suppression function. A significant correlation was observed between inflammatory cytokines, such as IL-6, IL-12 and TNFα, and the frequency of nTreg cells. Furthermore, the CD45RA^+^FoxP3^low^ nTreg cell subset increased when cultured with SLE serum compared to healthy donor serum, suggesting that the elevated inflammatory cytokines of SLE serum may promote nTreg cell proliferation/expansion.

**Conclusions/Significance:**

Our results indicate that impaired numbers of functional CD45RA^+^FoxP3^low^ naive Treg cell and CD45RA^−^FoxP3^low^ non-suppressive T cell subsets in inflammatory conditions may contribute to SLE development. Therefore, analysis of subsets of FoxP3^+^ T cells, using a combination of FoxP3, CD25 and CD45RA, rather than whole FoxP3^+^ T cells, will help us to better understand the pathogenesis of SLE and may lead to the development of new therapeutic strategies.

## Introduction

Human regulatory T cells (Treg cells) play an important role in T cell homeostasis and are critical regulators of immune tolerance [1]. Quantitative and/or qualitative deficiencies in Treg cells could lead to the development of autoimmune diseases [2], [3], [4]. Systemic lupus erythematosus (SLE) is a systemic autoimmune disease that is characterized by the presence of autoantibodies and immune complexes that target multiple organ systems. A deficiency in Treg cells results in the development of lupus-like characteristics, including glomerulonephritis and the development of DNA-specific antibodies, which might indicate failure of Treg cell-mediated suppression [5], [6], [7], [8], [9].

Many studies have assayed the number of Treg cells in the peripheral blood of SLE patients [10]. However, the reports on Treg cell numbers and function in SLE patients have been contradictory [11]. The lack of Treg-specific markers presents a challenging problem for the isolation and analysis of Treg cells. Importantly, FoxP3^+^ Treg cells may not be a functionally homogenous population. FoxP3 is constitutively expressed by Treg cells and is also induced in activated T cells [12], [13], [14]. This may explain the inconsistent results regarding the number of CD4^+^FoxP3^+^ cells reported for SLE patients. More recently, two groups reported that the percentage of CD4^+^CD25^+^FoxP3^+^ Treg cells is normal, whereas CD4^+^CD25^−^FoxP3^+^ T cells are significantly and consistently increased in patients with new onset SLE [15], [16]. However, those CD4^+^FoxP3^+^ cells in SLE patients are CD25^low^ or CD25^−^, and their identity has not been characterized. Whether subpopulations of FoxP3^+^ T cells are functionally different or reliably delineated is not known. In addition, how such subsets differ in proportion or function in the development of SLE remains to be determined. To characterize functionally heterogeneous CD4^+^FoxP3^+^ T cells, we employed CD45RA as a delineating surface marker for CD4^+^FoxP3^+^ cell subpopulations in SLE patients. It has been reported that some FoxP3^+^ cells are phenotypically naive (CD45RA^+^) in peripheral blood and exhibit a suppressive function, whereas other FoxP3^+^ cells phenotypically resemble memory T cells (CD45RA^−^) [4]. Miyara et al. have shown that human FoxP3^+^CD4^+^ T cells can be separated into three functionally and phenotypically unique subpopulations, based on the expression of FoxP3 and their cell surface phenotype [17]. The three distinct subpopulations are as follows: (1) CD45RA^+^FoxP3^low^ naive Treg cells (nTreg cells), (2) CD45RA^−^FoxP3^high^ activated Treg cells (aTreg cells), both of which are suppressive in vitro; and (3) non-suppressive cytokine-secreting CD45RA^−^FoxP3^low^ T cells (non-Treg cells). It has also been documented that the relative proportions of FoxP3^+^ T cell subpopulations show changes in dynamics in autoimmune diseases and Treg cell differentiation [4]. For instance, previously conflicting results may be interpreted as a change in circulating CD45RA^+^ nTreg cells into CD45RA^−^ aTreg. Furthermore, investigation of CD45RA expression on FoxP3^+^ cells might provide a consensus on the functional status of Treg cells in the development of SLE.

The development of SLE is also related to cytokine dysregulation. Cytokines assume a critical role in the differentiation, maturation and activation of T cells during SLE pathogenesis. In our previous study and in reports from other groups, proinflammatory cytokines, such as IL-1, IL-6, TNFα and cyclooxygenase-2 (COX-2), have been found to promote Treg proliferation/expansion, and to also support the proliferation of effector T cells (Teffs) [18], [19]. In addition, these cytokines have been shown to make Teffs relatively resistant to suppression by Treg cells [20], [21]. Not previously described, however, is a cytokine that can preferentially promote the activation of Teffs, while inhibiting Treg cell expansion. Whether the elevated levels of proinflammatory cytokines in SLE patients also contributes to the late generation of Treg cells is still unknown.

Here we report the characterization of the CD4^+^FoxP3^+^ T cell subpopulation in SLE development by using CD4, CD25 and CD45RA as surface markers of expression, thereby avoiding FoxP3^+^ non-Treg cells. We also explore the correlation with FoxP3, and determine its influence on Treg cell function, especially in the more activated stage of SLE. Furthermore, our data show that the high frequency of CD4^+^CD45RA^+^FoxP3^low^ nTreg cells is associated with the production of proinflammatory cytokines, including IL-6, IL-12 and TNFα, but not IL-17.

## Results

### Lack of correlation between FoxP3 and CD25 in CD4^+^ T cells of SLE patients

**Figure 1:**
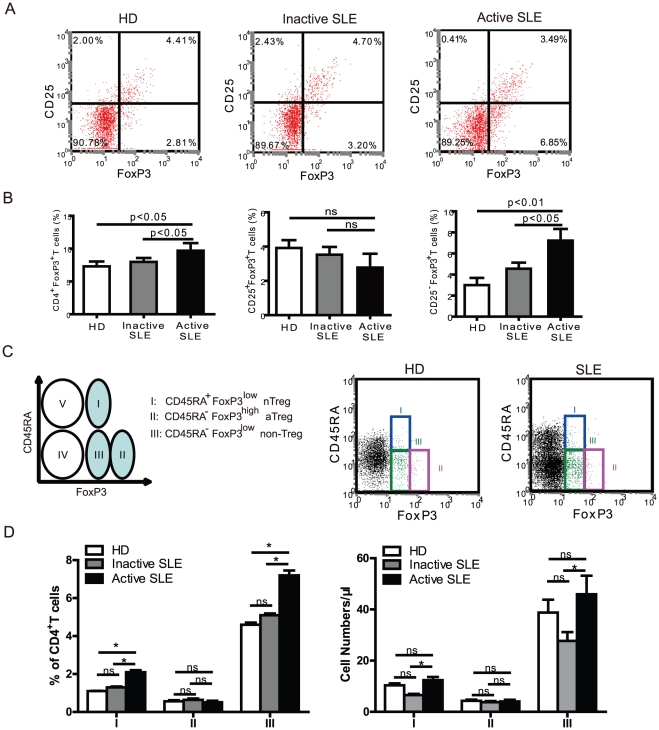
Phenotypic characterization of CD4^+^FoxP3^+^ T subset cells in patients with systemic lupus erythematosus (SLE). (A) PBMCs were gated on CD4^+^ T cells and analyzed for CD25 and FoxP3 expression by FCM. Data are representative of 5 independent experiments from healthy donors, and patients with inactive and active SLE. (B) Percentages of CD4^+^FoxP3^+^ T cells, CD4^+^CD25^−^FoxP3^+^ T cells and CD4^+^CD25^+^FoxP3^+^ T cells among CD4^+^ T cells in healthy donors (n=15), inactive SLE (n=26) and active SLE patients (n=15). Data represent the mean ± SEM. ns, means no significant differences. (C) Five subsets of CD4^+^ T cells are defined by the expression of CD45RA and FoxP3: CD45RA^+^FoxP3^low^ cells (I); CD45RA^−^FoxP3^high^ cells (II); CD45RA^−^FoxP3^low^ cells (III); CD45RA^−^FoxP3^−^ cells (IV); CD45RA^+^FoxP3^−^ (V) cells. Representative dot plots are shown for a healthy donor and an active SLE patient. (D) The percentages and absolute numbers of different CD4^+^ T cell fractions I, II, and III were analyzed in healthy donors (n=15), inactive SLE (n=26) and active SLE patients (n=15). Data represent mean ± SEM. ns, means no significant differences. *, mean p<0.05.

We compared the numbers of Treg cells in the peripheral blood from SLE patients and healthy donors by analysing the expression of FoxP3, a transcription factor critical to the development of Treg cells. When detecting distinct subsets of FoxP3^+^ T cells, based on the expression of CD25, there were considerably higher numbers of FoxP3^+^ cells that were CD25 dull or negative present in active SLE patients as compared to inactive SLE patients and healthy controls (Fig. 1A). There was a significant increase in the percentage of CD4^+^FoxP3^+^ cells in active SLE patients compared with either inactive SLE patients or healthy donors (Fig. 1B, left). In addition, the proportion of CD25^+^FoxP3^+^ cells was comparable among healthy donors (3.9±0.4%), inactive (3.7±0.5%) and active SLE (3.0±0.9%) (Fig. 1B, middle). In contrast, a significant increase in the mean percentage of CD25-FoxP3^+^ CD4^+^ T cells was observed in patients with active SLE (7.4±0.8%) when compared with that of normal controls (3.2±0.4%, p<0.01), or that of SLE patients with inactive disease (4.4±0.4%, p<0.05) (Fig. 1B, right), suggesting that in active SLE patients the CD4^+^FoxP3^+^ T cells are not correlated with the number of CD4^+^CD25^+^ T cells. There was no significant difference in the proportion of CD4^+^CD25^−^FoxP3^+^ cells between healthy donors and inactive SLE patients, although there was a trend toward an increase in CD4^+^CD25^−^FoxP3^+^ cells in the latter. The FoxP3^+^ subset of CD4^+^CD25^+^ cells was comparable in these three groups, suggesting that FoxP3 expression in CD4^+^ T cells from active SLE patients is dissociated from the CD4^+^CD25^+^ phenotype and, at least to some extent, reflects the activation of CD4^+^ T cells.

### Increased CD45RA^+^FoxP3^low^ and CD45RA^−^FoxP3^low^ T cell subsets in active SLE patients

Because of the different levels of FoxP3 expression with CD45RA staining as shown in Fig. 1C, we could separate three distinct CD4^+^FoxP3^+^ T subpopulations with a precise phenotype: CD45RA^+^FoxP3^low^ naive Treg cells (nTreg cells); CD45RA^−^FoxP3^high^ activated Treg cells (aTreg cells); and CD45RA^−^FoxP3^low^ (non-Treg cells). We observed a significant increase in CD45RA^+^FoxP3^low^ and CD45RA^−^FoxP3^low^ subsets in CD4^+^ T cells in patients with active SLE (2.1±0.4%; 7.2±1.0% respectively) compared with values found in healthy donors (1.3±0.2%; 4.6±0.6% respectively, p<0.05) or in patients with inactive SLE (1.1±0.1%; 5.1±0.4% respectively, p<0.05) (Fig. 1D, subpopulations Fr I and Fr III). However, there was no significant difference in CD45RA-FoxP3^high^ cells between the groups. The absolute numbers of this T cell subset was also supported for increased the subpopulations Fr I and Fr III(Fig. 1D right). The significant increase in CD45RA^+^FoxP3^low^ T cells and CD45RA^−^FoxP3 ^low^ T cells, and identification of the distinct subpopulations of FoxP3^+^ cells by CD45RA in patients with active SLE will provide us with information on the functional status of CD4^+^FoxP3^+^ T cells during SLE development.

### Correlation of CD45RA subsets and CD25-defined Treg cells

**Figure 2:**
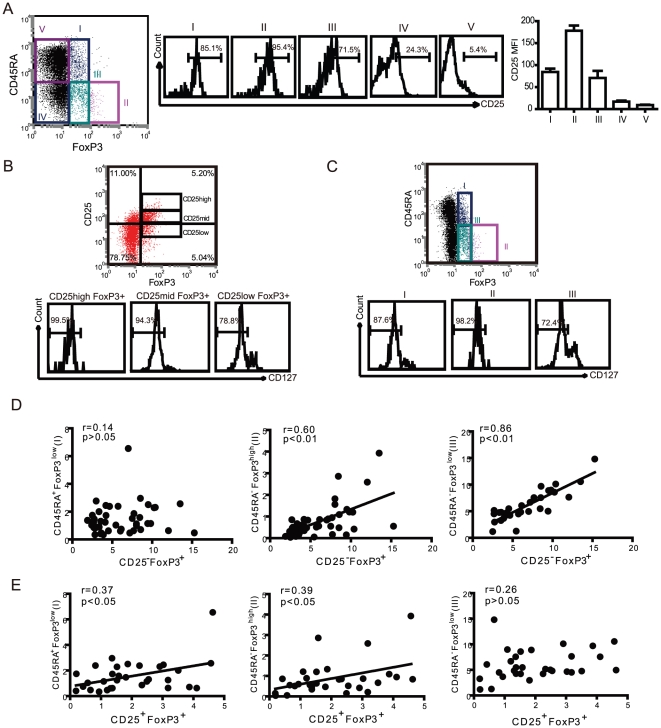
The relationship between the CD45RA/FoxP3-definded subsets of T cells and CD25/FoxP3-defined Treg cells in SLE. (A) The proportion of CD45RA^+^ FoxP3^low^ (I), CD45RA^−^FoxP3^high^ (II), CD45RA^−^FoxP3^low^ cells (III), CD45RA^−^FoxP3^−^ cells (IV) and CD45RA^−^FoxP3^−^ cells (V) were analyzed in relation to CD25 expression in active SLE patients. Mean Fluorescence Intensity (MFI) was indicated in different CD4^+^ T cell subpopulation. Data are representative of 5 independent experiments. (B) Expression of CD127 on CD25^high^, CD25^mid^, CD25^low^ subpopulation of CD4^+^FoxP3^+^ T cell from patients with active SLE. Data are representative of 5 independent experiments. (C) Expression of CD127 on CD45RA^+^FoxP3^low^ (I), CD45RA^−^FoxP3^high^ (II), CD45RA^−^FoxP3^low^ cells (III) subpopulation of CD4^+^FoxP3^+^ T cell from patients with active SLE. Data are representative of 5 independent experiments. (D) The correlation between CD45RA^+/−^FoxP3^+^ and CD25^−^FoxP3^+^ Treg cells in SLE patients (n=31). The solid line shows the regression curve. (E) The correlation between CD45RA^+/−^FoxP3^+^ and CD25^+^FoxP3^+^ Treg cells in SLE patients (n=31). The r value represents the calculated regression coefficient. The p value indicates the correlation index between the groups.

In patients with active and inactive SLE, T cells have been reported to display an activated phenotype through various markers for activated T cells [10]. To address whether different expression levels of FoxP3 in patients with active SLE might be related to their T cell activation phenotype, CD4^+^FoxP3^+^ T cells were analyzed for their expression of CD45RA. As shown in Fig. 1, significantly higher proportions of FoxP3^+^ T cells were observed in CD45RA^+^FoxP3^low^ and CD45RA^−^FoxP3^low^ subsets in patients with active SLE. We focused on the correlation of these two populations with CD25^−^FoxP3^+^ T cells. Comparative analyzes were performed in patients with active SLE. As shown in Fig. 2A, CD25 expression is different in CD45RA/FoxP3-defined T cell subsets. The CD45RA^−^FoxP3^high^ (Fr II) subset contains higher percentage and intensity of CD25 positive cell. In contrast, the Fr I and III subsets T cell express medium or low CD25. We gated CD127^low/−^ expression on CD4^+^Foxp3^+^ T cells, dependent on CD25 or CD45RA, respectively. We found that all CD4^+^Foxp3^+^ T cells had a low expression level of CD127, and percentages of CD127^low/−^ in CD25^high^ and CD25^mid^ were higher than that in CD25^low^ subpopulations of CD4^+^Foxp3^+^ T cells (Fig. 2B). Furthermore, the expressions of CD127^low/−^ on CD45RA/FoxP3 defined T cells subpopulation showed significant differences in CD25high, CD25low, and CD25- subpopulations (Fig. 2C). CD127^low/−^ expressions were high in CD45RA^−^FoxP3^high^ (II) but low in CD45RA^+^FoxP3^low^ (I) and CD45RA^−^FoxP3^low^ (III) subpopulations. This result suggested that CD127^low/−^ was not a perfect candidate surface marker for intracellular FoxP3 in CD4+CD25- T cells in patients with SLE. We then analyzed this correlation, and our results show that CD45RA^−^FoxP3^low^ T cells were significantly correlated to the CD25^−^FoxP3^+^ subset (Fig. 2D). In addition, the proportions of CD45RA^+^FoxP3^low^ T cells were significantly correlated to the proportions of CD25^+^FoxP3^+^ T cells, suggesting that the CD45RA^+^FoxP3^low^ subset may be naive Treg cells that have suppressive capability (Fig. 2E). In contrast, there was no correlation between proportions of CD45RA^+^FoxP3^low^ and CD25^−^FoxP3^+^ T cells. These findings further strengthened our confidence that CD45RA may be used as a marker to sort FoxP3^+^ T cells in a CD4^+^ T cell population.

### Correlation of CD45RA subsets in SLE development

**Figure 3:**
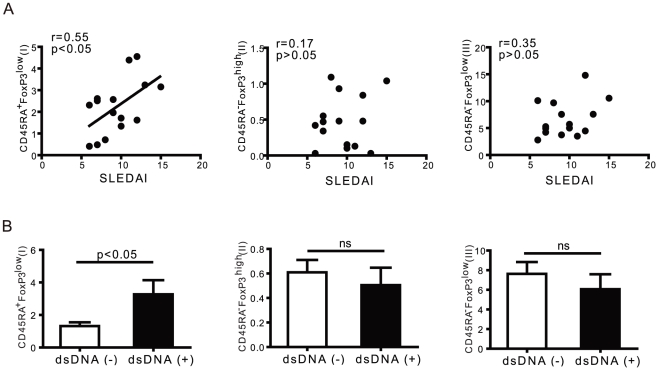
The relationship between the CD45RAFoxP3 subset of T cells and clinical disease activity. Three subsets of FoxP3^+^ T cells were analyzed for clinical correlation. (A) A positive correlation between the proportion of CD45RA^+^FoxP3^low^ (I) cells and clinical severity of the flare, scored using the SLEDAI in active SLE patients (n=15). (B) A significant difference was observed for proportions of CD45RA^+^FoxP3^low^ (I) cells in the status of anti-dsDNA antibody in active SLE patients (n=15). Data represent the mean ± SEM.

To determine whether the appearance of CD45RA subsets is linked to clinical disease, we analyzed the correlation of CD45-defined FoxP3^+^ T cell subsets with SLE disease activity scores. The CD45RA subset was also compared with status of anti-dsDNA antibody in patients. As described above, significantly higher proportions of CD45RA^+^FoxP3^low^ nTreg cells and CD45RA^−^FoxP3^low^ non-Treg cells were observed in PBMCs from patients with active SLE as compared to healthy donors. The most noteworthy finding is that a significant correlation was found for populations of CD45RA^+^FoxP3^low^ T cells with the SLEDAI score (r=0.55, p<0.05) (Fig. 3A). We examined the presence of anti-dsDNA using an ELISA test, then separated the patients into two groups that were anti-dsDNA positive (5 cases) or anti-dsDNA negative (10 cases). Regarding the subsets of Treg cells, the percentage of CD45RA^+^FoxP3^low^ regulatory T cells was significantly higher in the anti-DNA positive group. This may be due to an impairment of the main suppressive function in this subset of rTregs, through an increase in the number of CD45RA^+^FoxP3^low^ regulatory T cells to overcome the control of the autoimmune response(Fig. 3B).

### Impairment of the suppressive function of the CD45RA^+^FoxP3^low^subset in patients with active SLE

**Figure 4:**
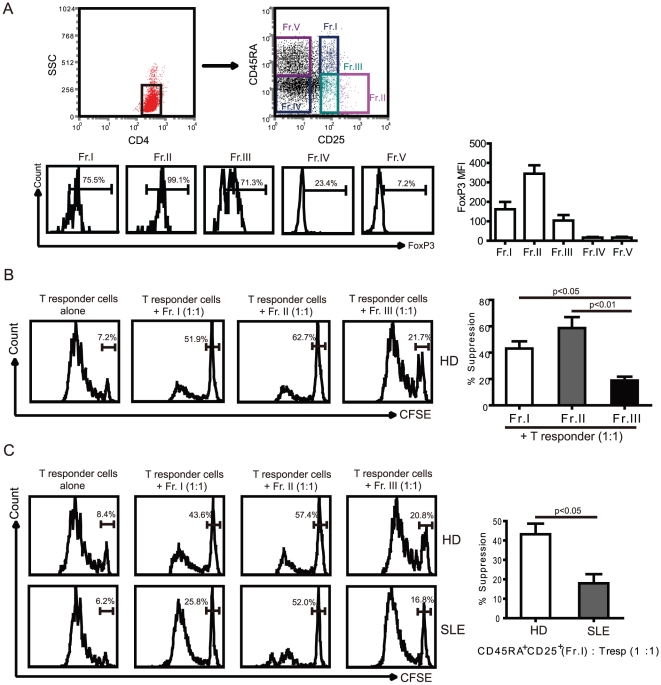
Functional characterization of the CD45RA/FoxP3-defined subset of T cells in SLE patients. (A) Five subsets of CD4^+^ T cells were defined by the expression of CD45RA and CD25: CD25^+^CD45RA^+^ cells (Fr. I); CD25^++^CD45RA^−^ cells (Fr. II); CD25^+^CD45RA^−^ cells (Fr. III); CD25^−^CD45RA^−^ cells (Fr. IV); and CD25^−^CD45RA^+^ cells (Fr. V). Expression of FoxP3 and MFI of FoxP3 were analyzed in CD25 and CD45RA defined T cell subsets. (B) CD4^+^CD25^−^CD45RA^+^ responder T cells were labelled with CFSE, and cultured at a 1∶1 ratio with unlabeled Fr.I, II, or III purified from healthy donor (n=3). Cell proliferation was assessed by CFSE dilution. The percent suppression is shown. Statistical analysis of the suppression percentage of T responder cells was performed by nonparametric Mann-Whitney U test. (C) Sorted CD4^+^CD25^−^CD45RA^+^ responder T cells were co-cultured with their autologous sorted Fr. I, II, or III, respectively, in order to assess the function of Fr. I/II/III cell suppression from healthy donors and active SLE patients. Representative FCM data are shown. Statistical analysis of the suppression percentage of T responder cells was performed from 5 healthy donors and 5 patients with active SLE.

Because the CD4^+^CD45RA^+^ subset may resemble Treg or activated T cells, according to the phenotypic analyzes described above, we next sought to determine their suppressive capacity in vitro. Due to the limitations of FoxP3 to separate human Treg cells for functional studies we utilized the phenotypic strategy described above. Specifically, for the characterization of functionally heterogeneous populations of CD4^+^FoxP3^+^ T cells and to avoid contamination with activated responder T cells, we performed a combination of CD25, FoxP3 and CD45RA staining of CD4^+^ T cells in peripheral blood lymphocytes from normal donors and patients with SLE. Similar to the FoxP3/CD45RA combination, combined CD25/CD45RA surface expression can also be used to identify the five fractions (Fig. 4A, up). Our results showed that FoxP3 expression is different in CD25/CD45RA-defined T cell subsets (Fig. 4A, down), which FoxP3 mainly expressed in fraction I, II, III subpopulations and fraction II Treg cell have higher MFI of FoxP3 than fraction I and fraction II. To assess the suppressive potency of each fraction, we first measured the extent of CFSE dilution of labelled CD25^−^CD45RA^+^ T responder cells cocultured with an equal number of a Treg fraction from healthy donors. Our results showed that fraction I and II potently suppressed proliferation of responder cells, whereas fraction III failed to inhibit the responder T cell proliferation. To investigate the function of increased nTreg cells in SLE patients, we measured and compared the function of CD25^+^CD45RA^+^ nTreg cells from healthy donor and active SLE patients (Fig. 4B). There were no significant differences in the suppressive activity of fraction II and III cells between HD and active SLE patients (Fig. 4C). However, CD45RA^+^FoxP3^low^ T cells (Fr. I) from patients with active SLE exhibited significantly less suppressive activity (17.3±7.0%) than fraction I cells from healthy donors (41.2±6.5%, p<0.05) (Fig. 4C). The suppressive rate of CD45RA^+^FoxP3^low^ T cells from patients with inactive SLE revealed a less striking, but significant decrease in the percent inhibition of Tresp cell proliferation, as compared with that of nTreg cells from healthy donors (data not show).

### Increase in the CD45RA^+^FoxP3^low^ T cell subset through proinflammatory cytokines, but not IL-17

**Figure 5:**
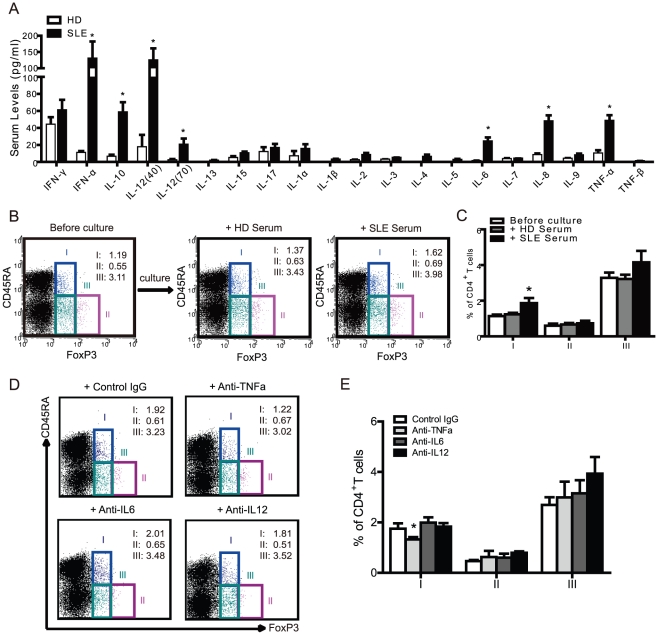
Proinflammatory cytokines contribute to CD45RA^+^FoxP3^+^ T cell development in SLE. (A) A series of cytokines were measured by high sensitivity multiplex assay from the sera of healthy donors (n=15) and active SLE patients (n=15). *p<0.05 compared to healthy sera. (B) Healthy isolated PBMCs were stimulated with the sera (1∶5 dilutions) from autologous healthy donors or active SLE patients, cultured for 3 days and analyzed for CD45RA and FoxP3 expression by FCM before and after culture. Representative FCM data was shown. (C) Statistical analysis of the percentage of each FoxP3^+^ subset among CD4^+^ T cells was performed before and after culture as defined in (B) from 5 healthy donors and 5 active SLE patients. *, p<0.05 compared to unstimulated control. (D) Healthy isolated PBMCs were stimulated by the sera (1∶5 dilutions) from active SLE patients with control IgG (10 µg/ml), anti-TNFa (10 µg/ml), anti-IL6 (10 µg/ml), anti-IL12 (10 µg/ml) respectively, cultured for 3 days and analyzed for CD45RA and FoxP3 expression by FCM. Representative FCM data was shown. (E) Statistical analysis of the percentage of each FoxP3^+^ subset among CD4^+^ T cells was performed as defined in (D) from 5 active SLE patients. *, p<0.05 compared to control IgG.

SLE is characterized by systemic inflammation. Therefore, the induction of excessive levels of proinflammatory cytokines might, in some way, promote or convert Treg cells. Through cytokine analysis, serum levels of IL-6, IL-8, IL-10, IL-12, IFNα and TNFα were significantly higher in active SLE patients than in healthy donors (Fig. 5A). As illustrated in Table 1, the frequencies of circulating CD45RA^+^FoxP3^low^ (I) T cell subsets were significantly and positively correlated with the levels of serum IL-1ß, IL-6, IL-12, TNFα and IL-2 compared to other cytokines in SLE patients, such as IFNα and IL-17. We found an increase in the CD45RA^+^FoxP3^low^ T cell subset in culture with SLE serum (1.9±0.1%) compared to healthy donor serum (1.2±0.1%, p<0.05), (Fig. 5B and C), which suggests that SLE serum contains proinflammatory cytokines that can induce or promote expansion of the CD45RA^+^FoxP3^low^ nTreg cells. Furthermore, we can block raising CD45RA^+^FoxP3^low^ T cells by using TNFα antibody(Fig. 5D). Which indicated that some specific cytokine contributed Treg cell development in SLE.

**Table 1:** The correlation of CD4^+^Foxp3^+^ T cell subsets with serum cytokines in patients with active SLE.

Cytokines	CD45RA^+^FoxP3^low^ T cell (I)		CD45RA^−^FoxP3^high^ T cell (II)		CD45RA^−^FoxP3^low^ T cell (III)	
	r	p	r	p	r	p
**IL-12**	0.91	<0.01*	0.50	0.10	0.22	0.49
**IL-6**	0.69	0.02*	0.16	0.62	−0.39	0.21
**IL-1β**	0.77	0.01*	−0.23	0.47	−0.22	0.49
**TNF-α**	0.69	0.02*	0.42	0.17	0.01	0.98
**IL-2**	0.84	0.01*	0.01	0.98	−0.29	0.37
**IFN-γ**	−0.20	0.51	−0.23	0.46	0.79	0.01*
**IL-17**	−0.22	0.50	0.03	0.94	−0.61	0.04*
**IFN-α**	−0.20	0.51	−0.23	0.46	−0.50	0.10
**IL-10**	0.28	0.38	0.19	0.56	−0.01	0.98

* p<0.05, Spearman's rank correlation tests.

## Discussion

The observations on the number and function of Treg cells in patients with SLE have, in many respects, been contradictory [22], [23], [24]. This may be due to the presence of contaminating active CD4^+^ non-Treg cells, which can transiently express FoxP3, resulting in a constant threat to Treg cell purity. However, there is currently a lack of defined markers that can identify Treg phenotypes that can replace the intracellular FoxP3 marker for use in subsequent functional studies of Treg cells. Here, we report increases in CD4^+^CD45RA^+^FoxP3^+^ and CD4^+^CD45RA^−^FoxP3^low^ cells in active SLE patients. The increased CD4^+^CD25^−^FoxP3^+^ T cells in SLE patients may have been derived from the CD4^+^CD45RA^−^FoxP3^low^ subset, which is a non-regulatory T cell population without suppressive function. Further studies of their suppressive function in vitro revealed that CD45RA^+^FoxP3^low^ T cells in active SLE failed to suppress responder cell proliferation. Furthermore, CD45RA^+^FoxP3^low^ subsets were increased in active SLE through the release of circulating cytokines, including IL-6, IL-12 and TNFα. Together, these results suggest that Treg cells identified by CD45RA along with CD25 and FoxP3 may indicate the disease activity in peripheral blood of human SLE patients.

In the current study, we found an increased population of CD45RA^+^FoxP3^low^ T cells in active SLE patients. However, there was no significant difference in the population of CD45RA^−^FoxP3^high^ T cells between patients with active SLE and healthy donors. Although CD45RA^−^FoxP3^low^ T cells can possibly be converted into CD45RA^−^FoxP3^high^ T cells, which are terminally differentiated and rapidly undergo apoptosis [25], the increased frequency of CD45RA^+^FoxP3^low^ is associated with defective suppressive function in vitro, as well as the development of DNA-specific antibodies in patients with active SLE. The abnormal suppressive function of Treg cells in SLE patients may be due to the resistance of SLE-associated effector T cells toward Treg-mediated suppression, or the indirect blockade of Treg cell effectiveness by antigen-presenting cells [26], [27] This scenario provides a rational explanation for the increase in peripheral blood CD45RA^+^FoxP3^low^ T cells, which correlated with the quantity of CD4^+^CD25^+^FoxP3^+^ Treg in active lupus, and may indicate a positive feedback response to the resistant/blockade effect on Treg cell suppression. This leads to the following question: Where do the increased circulating CD4^+^CD25^+^FoxP3^+^ Treg cells originate? Most of the CD4^+^CD25^+^FoxP3^+^ T cells in the peripheral blood from patients with SLE have undergone autoantigen stimulation and thus are derived from the memory CD4^+^ T cell compartment. To limit interference by CD45RA^+^Foxp3T cell number with age, we selected age-matched patients and healthy controls. Patients and controls aged about 30–35 years old were enrolled in this study. Although rTreg cells were decreased in aged donors, which has been reported by Miyara et al [17], we observed a high proportion of rTreg among CD4^+^ T cells in active SLE patients compare to age-matched healthy controls. There may be many activated T cells in SLE patients or younger donors. Most of the Foxp3^hi^ aTregs may be derived from activated and proliferating rTreg cells, and a small fraction of aTreg cells derived from Foxp3^−^CD4^+^ non-Treg cells in vivo. No significant increase in aTreg cells was observed in our SLE patients, but we did find a significant increase in rTreg and Foxp3^−^CD4^+^ non-Treg cells in the patients with active SLE, which was consistent with the recent report by Miyara et al [17]. Thus, the lower suppressive capacity seen in coculture assays of CD45RA^+^FoxP3^low^T cells from patients with active SLE could be attributed either to an impaired suppressor function of the Fr.I nTreg cells or to a resistance of Tresp cells to Treg cell-mediated suppression. Moreover, despite impaired nTreg cells suppressive function, the function of Tresp cells resistance to Treg cells is not entirely clear. One further consideration is that CD25^+^CD45RA^−^ T cells are not equivalent in number to CD45RA^−^FoxP3^low^ non-Treg cells in patients with SLE because of the increase in CD4^+^CD25^−^FoxP3^+^ T cells. With regard to Tresp cells becoming relatively resistant to suppression by Treg cells through the expression of specific cytokines, our in vitro suppressive function assay using isolated Treg and Tresp cells suggest that this is not the case. Nonetheless, the presence of proinflammatory cytokines in vivo or in vitro may lead to abrogated Treg cell suppressor function. In humans, it has been suggested that memory CD4^+^ T cells may be the peripheral origin of adaptive CD4^+^CD25^+^FoxP3^+^ cells, also called inducible Treg cells [28], [29]. Our current data provide evidence suggesting that the variation in CD4^+^CD25^+^CD45RA^+^ T cell frequency may result from a defect in function or quality of these cells in active SLE.

More recently, several groups have reported that there is a considerable number of CD4^+^CD25^−^FoxP3^+^ T cells present in the peripheral blood of patients with SLE [11]. Therefore speculations on increased or decreased proportions of Treg cells, based on phenotypic analysis in patients with SLE, must be made with caution, as increased proportions of activated T cells can be observed under conditions of chronic inflammatory diseases [30] and might contaminate assays relying of gated populations of Treg cells. This prompted us to ask whether CD4^+^CD25^−^FoxP3^+^ T cells in SLE patients represent activated T cells or Treg. We performed detailed comparative phenotypic analyzes of CD4^+^CD25^+^FoxP3^+^ and CD4^+^CD25^−^FoxP3^+^ from SLE patients and healthy controls, and used a combination of CD45RA surface markers to substitute for FoxP3, which allowed us to isolate these cells for functional studies. Using the CD45RA marker, we identified that the population of CD4^+^CD25^−^FoxP3^+^ most likely contained the CD45RA^+^FoxP3^low^ and CD45RA^−^FoxP3^low^ subsets. The CD45RA^+^FoxP3^high^ population was not significantly different between HD and active SLE, although it was positively correlated to the CD25^+^FoxP3^+^ subsets, suggesting that CD25^+^FoxP3^+^ may be not an exclusive marker of Treg cells in SLE, as it may be expressed in active T cells. The CD45RA^−^FoxP3^low^ T cell population was not associated with suppressive function in our comparison study between SLE patients and healthy donors. Based on our data, we conclude that CD4^+^CD25^−^FoxP3^+^ T cells in SLE patients share several properties with conventional Treg, but are otherwise distinct in certain functional qualities.

As SLE is characterized by systemic inflammation, the induction of excessive levels of proinflammatory cytokines [31] may “prime" the Tresp cells to develop resistance to Treg cell-mediated suppression, as seen in our suppressor assays. The inflammatory cytokines of the local environment might be responsible for this effect. These cytokines could be produced by the Tresp cells themselves, in an autocrine manner, or be produced by other cell types within the inflammatory environment. The abrogation of Treg cell function in suppressor assays in the presence of proinflammatory cytokines such as IL-1 [21], TNFα [32], IL-6 [33], and IL-12 [34] in mice or humans, and the high levels of Treg cells in human SLE, have led us to initiate studies on the role of these cytokines. Our results show that proinflammatory cytokine production is significantly increased in the serum of patients with active SLE, including IL-6, TNFα and IL-12, and these cytokines were positively correlated with CD45RA^+^FoxP3^low^ T cells. Furthermore, we were able to induce CD45RA^+^FoxP3^low^ T cells from naïve T cell by using those sera that contained proinflammatory cytokines from SLE patients. In our experiments, we can block the increase in CD45RA^+^FoxP3^low^ T cells by using TNFα antibody, which indicates that there is a specific cytokine contribution to Treg cell development in SLE. Although characteristics of the SLE marker IFNα were elevated in SLE patients [35], this was not associated with nTreg cells. The contribution of type I interferon to the development of SLE has been a long-standing interest of Shen and other groups. More recent data from Shen and others have addressed elevated levels of IFNα by antigen-presenting cells (APCs) from patients with active SLE, which inhibited Treg cell suppressive function [27]. We also found nTreg cells from patients with active SLE with a defective suppression function. Whether the defect in those subsets of Treg cells is mediated by IFN needs to be studied further. We also report that the increased CD45RA^+^FoxP3^low^ subset in active SLE was linked to a lack or abrogation of suppressive function, which further supports the finding that abnormally increased nTreg cells in active SLE are unable to suppress the systemic immune response and proinflammatory cytokine production. Future studies will need to evaluate the mechanisms by which proinflammatory cytokines generate Tresp cell resistance. It is already known that hyperactivation of certain intracellular signaling pathways, such as the phosphatidylinositol 3-kinase/Akt pathway, can contribute to this process in different genetically engineered mouse models that are prone to developing autoimmune diseases [36].

In summary, we have identified an increase in the subset of CD45RA^+^FoxP3^low^ T cells that contributes to SLE progression with aberrant inflammatory status. We propose that the combination of CD25 and CD45RA is therefore the best set of markers for identifying human FoxP3^+^ Treg cells as nTreg and aTreg cells. This distinction is highly informative in assessing the dynamics of Treg cell differentiation under physiological and disease conditions. Impaired functions and numbers of CD45RA^+^FoxP3^low^ nTreg cells and CD45RA^−^FoxP3^low^ non-Treg cell subsets in inflammatory conditions may contribute to SLE development. By analyzing a subset of FoxP3^+^ T cells, rather than whole FoxP3^+^ T cell population, may help us to better understand the pathogenesis of SLE and support the development of novel therapeutic strategies.

## Materials and Methods

### Patients and controls

Forty-one SLE patients (mean ± SD age 32.7±7.4 years) who met the revised SLE criteria of the American College of Rheumatology [37], [38] and fifteen age- and sex-matched healthy donors (HD) (mean ± SD age 30.4±5.9 years) were enrolled in this study. In our study, we only included the patients who were not receiving drugs at the time of the study. Prior to participation, written informed consent was obtained from all subjects. All studies were performed in accordance with the Declaration of Helsinki. The study was approved by the Research Ethics Board of Xinhua Hospital, Shanghai Jiao Tong University School of Medicine. All patients were diagnosed as having SLE according to the revised SLE Disease Activity Index (SLEDAI), and a score ≥5 defined active diseases. According to their disease activity, patients were divided into two groups. One group comprised 15 patients with active SLE (median SLEDAI score 10 [range 6–15]), and a second group comprised 26 patients with inactive SLE (median SLEDAI score 2 [range 0–4]). Healthy donors had no acute or chronic inflammatory or infectious disease, ongoing thrombosis, or neoplasia. The following were measured in the SLE patients in the clinical immunology laboratory of the Xinhua Hospital: differential white cell count; anti-double-stranded DNA (dsDNA); immunoglobulin G (IgG); and the levels of plasma complement C3 and C4, and urine protein. These tests were all performed in parallel with the analysis of CD4^+^FoxP3^+^ T cell subpopulations.

### Phenotypic analysis

Peripheral blood mononuclear cells (PBMCs) were freshly isolated by Ficoll-Hypaque density gradient centrifugation. PBMCs were re-suspended in PBS supplemented with 2% bovine serum albumin at a concentration of 1×10^6^ cells/ml. Fluorochrome-labeled mouse anti-human monoclonal antibodies targeted against CD45RA-PE, CD3-ECD, CD25-PC5 and CD4-PC7 were purchased from Beckman Coulter. FoxP3-FITC and IgG2a-FITC (eBioscience) were used, together with appropriate isotype controls, to permit the identification of positive and negative cell populations. Intracellular FoxP3 staining was performed according to the manufacturer's instructions. Multiple-color flow cytometry (FCM) analysis was performed using a BD FACS Aria flow cytometer (BD). For cell sorting, peripheral blood was collected from either healthy donors or SLE patients, and PBMCs were isolated and stained with the relevant fluorochrome-labeled monoclonal antibodies. The FACS Aria Flow cytometer was adjusted with Accudrop (BD) for optimum sorting conditions. The purity of sorted cells was verified after sorting and was >95%.

### Functional assays

PBMCs from SLE patients or healthy donors were isolated and sorted into CD4^+^CD25^+^CD45RA^+^, CD4^+^CD25^high^CD45RA^−^, CD4^+^CD25^+^CD45RA^−^, and CD4^+^CD25^−^CD45RA^+^ subpopulations by FCM. Purity of each sorted population was consistently >95%. The CD25^high^ gate was adjusted to contain CD4^+^ T cells that express CD25 more brightly than CD4^+^CD25^+^ cells. RPMI 1640 medium supplemented with 10% fetal bovine serum, 100 IU/ml penicillin, and 100 mg/ml streptomycin (Sigma) was used for T cell cultures. For the carboxyfluorescein diacetate succinimidyl ester (CFSE) dilution assay, CD4^+^CD25^−^CD45RA^+^ responder T (Tresp) cells were labeled with 1 µmol/ml CFSE (Invitrogen) for 8 min at 37°C. 1×10^4^ CD4^+^CD25^−^CD45RA^+^ T cells were co-cultured in the presence or absence of sorted CD4^+^ subsets cells for assessing their suppressive capacity at 1∶1. Subsequently, mixed T cell cultures were stimulated with anti-CD3/CD28 coated beads (Dynal Biotech) in 96-well plates for 4 days. Proliferation of CFSE-labeled cells was analyzed by flow cytometry either immediately or after 4 days.

### Serum stimulation assay

Healthy PBMCs were stimulated with serum (1∶5 dilutions) from healthy donors or active SLE patients, cultured for 3 days and analyzed by FCM before and after culture, as described above.

### Cytokine assays

Sera derived from patients with SLE and healthy donors were collected and frozen at −70°C until used. Serum cytokine levels were measured using an immunobead-based 20-plex assay (Luminex) and included the following cytokines: IL-1α, IL-1β, IL-2, IL-3, IL-4, IL-5, IL-6, IL-7, IL-8, IL-9, IL-10, IL-12(p40), IL-12(p70), IL-13, IL-15, IL-17, IFNγ, IFNα, TNFα and TNFβ. Panels of capture antibody-coated beads and labeled detection antibodies were purchased from Millipore, which were pretested and qualified by the manufacturer to ensure the absence of cross-reactivity. The assay sensitivity varied from 5 to 15 pg/ml depending on the analysis. The assays were performed using the Luminex 200 System (Luminex).

### Statistical analysis

Analysis of the data was performed using GraphPad Prism version 5.0 for Windows (GraphPad Software, San Diego, CA, USA). Values are expressed as mean ± SEM unless otherwise indicated. The Kolmogorov-Smirnov test was used to evaluate the distribution of each parameter. For data with normal distribution and homogeneity of variance, an independent-sample *t* test was used to compare differences between two groups, and a one-way ANOVA with Tukey's post-hoc test was performed if there were three or more means. The Mann-Whitney test was used to compare data with a non-normal distribution. Correlations were assessed by Spearman's rank correlation coefficients and significance was determined after a Bonferroni correction. A value of *p*<0.05 was considered significant in all statistical tests.

## References

[1] Sakaguchi S (2008). Regulatory T cells in the past and for the future.. Eur J Immunol.

[2] Sakaguchi S, Sakaguchi N, Asano M, Itoh M, Toda M (1995). Immunologic self-tolerance maintained by activated T cells expressing IL-2 receptor alpha-chains (CD25). Breakdown of a single mechanism of self-tolerance causes various autoimmune diseases.. J Immunol.

[3] Bonelli M, Savitskaya A, von Dalwigk K, Steiner CW, Aletaha D (2008). Quantitative and qualitative deficiencies of regulatory T cells in patients with systemic lupus erythematosus (SLE).. Int Immunol.

[4] Buckner JH (2010). Mechanisms of impaired regulation by CD4(+)CD25(+)FOXP3(+) regulatory T cells in human autoimmune diseases.. Nat Rev Immunol.

[5] Hahn BH (1998). Antibodies to DNA.. N Engl J Med.

[6] Zhang B, Zhang X, Tang FL, Zhu LP, Liu Y (2008). Clinical significance of increased CD4+CD25-Foxp3+ T cells in patients with new-onset systemic lupus erythematosus.. Ann Rheum Dis.

[7] La Cava A (2008). T-regulatory cells in systemic lupus erythematosus.. Lupus.

[8] Hahn BH, Anderson M, Le E, La Cava A (2008). Anti-DNA Ig peptides promote Treg cell activity in systemic lupus erythematosus patients.. Arthritis Rheum.

[9] Chavele KM, Ehrenstein MR (2011). Regulatory T-cells in systemic lupus erythematosus and rheumatoid arthritis.. FEBS Lett.

[10] Gerli R, Nocentini G, Alunno A, Bocci EB, Bianchini R (2009). Identification of regulatory T cells in systemic lupus erythematosus.. Autoimmun Rev.

[11] Horwitz DA (2008). Regulatory T cells in systemic lupus erythematosus: past, present and future.. Arthritis Res Ther.

[12] Tran DQ, Ramsey H, Shevach EM (2007). Induction of FOXP3 expression in naive human CD4+FOXP3 T cells by T-cell receptor stimulation is transforming growth factor-beta dependent but does not confer a regulatory phenotype.. Blood.

[13] Wang J, Ioan-Facsinay A, van der Voort EI, Huizinga TW, Toes RE (2007). Transient expression of FOXP3 in human activated nonregulatory CD4+ T cells.. Eur J Immunol.

[14] Simonetta F, Chiali A, Cordier C, Urrutia A, Girault I (2010). Increased CD127 expression on activated FOXP3+CD4+ regulatory T cells.. Eur J Immunol.

[15] Bonelli M, Savitskaya A, Steiner CW, Rath E, Smolen JS (2009). Phenotypic and functional analysis of CD4+ CD25- Foxp3+ T cells in patients with systemic lupus erythematosus.. J Immunol.

[16] Yang HX, Zhang W, Zhao LD, Li Y, Zhang FC (2009). Are CD4+CD25-Foxp3+ cells in untreated new-onset lupus patients regulatory T cells?. Arthritis Res Ther.

[17] Miyara M, Yoshioka Y, Kitoh A, Shima T, Wing K (2009). Functional delineation and differentiation dynamics of human CD4+ T cells expressing the FoxP3 transcription factor.. Immunity.

[18] Chen X, Baumel M, Mannel DN, Howard OM, Oppenheim JJ (2007). Interaction of TNF with TNF receptor type 2 promotes expansion and function of mouse CD4+CD25+ T regulatory cells.. J Immunol.

[19] Yuan XL, Chen L, Li MX, Dong P, Xue J (2010). Elevated expression of Foxp3 in tumor-infiltrating Treg cells suppresses T-cell proliferation and contributes to gastric cancer progression in a COX-2-dependent manner.. Clin Immunol.

[20] Pasare C, Medzhitov R (2003). Toll pathway-dependent blockade of CD4+CD25+ T cell-mediated suppression by dendritic cells.. Science.

[21] O'Sullivan BJ, Thomas HE, Pai S, Santamaria P, Iwakura Y (2006). IL-1 beta breaks tolerance through expansion of CD25+ effector T cells.. J Immunol.

[22] Valencia X, Yarboro C, Illei G, Lipsky PE (2007). Deficient CD4+CD25high T regulatory cell function in patients with active systemic lupus erythematosus.. J Immunol.

[23] Miyara M, Amoura Z, Parizot C, Badoual C, Dorgham K (2005). Global natural regulatory T cell depletion in active systemic lupus erythematosus.. J Immunol.

[24] Horwitz DA (2010). Identity of mysterious CD4+CD25-Foxp3+ cells in SLE.. Arthritis Res Ther.

[25] Booth NJ, McQuaid AJ, Sobande T, Kissane S, Agius E (2010). Different proliferative potential and migratory characteristics of human CD4+ regulatory T cells that express either CD45RA or CD45RO.. J Immunol.

[26] Vargas-Rojas MI, Crispin JC, Richaud-Patin Y, Alcocer-Varela J (2008). Quantitative and qualitative normal regulatory T cells are not capable of inducing suppression in SLE patients due to T-cell resistance.. Lupus.

[27] Yan B, Ye S, Chen G, Kuang M, Shen N (2008). Dysfunctional CD4+,CD25+ regulatory T cells in untreated active systemic lupus erythematosus secondary to interferon-alpha-producing antigen-presenting cells.. Arthritis Rheum.

[28] Prakken BJ, Samodal R, Le TD, Giannoni F, Yung GP (2004). Epitope-specific immunotherapy induces immune deviation of proinflammatory T cells in rheumatoid arthritis.. Proc Natl Acad Sci U S A.

[29] Belkaid Y, Piccirillo CA, Mendez S, Shevach EM, Sacks DL (2002). CD4+CD25+ regulatory T cells control Leishmania major persistence and immunity.. Nature.

[30] Isomaki P, Clark JM, Panesar M, Cope AP (2005). Pathways of T cell activation and terminal differentiation in chronic inflammation.. Curr Drug Targets Inflamm Allergy.

[31] Wong CK, Ho CY, Li EK, Lam CW (2000). Elevation of proinflammatory cytokine (IL-18, IL-17, IL-12) and Th2 cytokine (IL-4) concentrations in patients with systemic lupus erythematosus.. Lupus.

[32] Valencia X, Stephens G, Goldbach-Mansky R, Wilson M, Shevach EM (2006). TNF downmodulates the function of human CD4+CD25hi T-regulatory cells.. Blood.

[33] Wan S, Xia C, Morel L (2007). IL-6 produced by dendritic cells from lupus-prone mice inhibits CD4+CD25+ T cell regulatory functions.. J Immunol.

[34] King IL, Segal BM (2005). Cutting edge: IL-12 induces CD4+CD25− T cell activation in the presence of T regulatory cells.. J Immunol.

[35] Tang Y, Luo X, Cui H, Ni X, Yuan M (2009). MicroRNA-146A contributes to abnormal activation of the type I interferon pathway in human lupus by targeting the key signaling proteins.. Arthritis Rheum.

[36] Barath S, Aleksza M, Tarr T, Sipka S, Szegedi G (2007). Measurement of natural (CD4+CD25high) and inducible (CD4+IL-10+) regulatory T cells in patients with systemic lupus erythematosus.. Lupus.

[37] Bombardier C, Gladman DD, Urowitz MB, Caron D, Chang CH (1992). Derivation of the SLEDAI. A disease activity index for lupus patients. The Committee on Prognosis Studies in SLE.. Arthritis Rheum.

[38] Hochberg MC (1997). Updating the American College of Rheumatology revised criteria for the classification of systemic lupus erythematosus.. Arthritis Rheum.

